# Investigative genetic genealogy practices warranting policy attention: Results of a modified policy Delphi

**DOI:** 10.1371/journal.pgen.1011520

**Published:** 2025-01-16

**Authors:** Christi J. Guerrini, Louiza Kalokairinou, Jill O. Robinson, Whitney Bash Brooks, Stephanie M. Fullerton, Sara Huston, Jacklyn Dahlquist, Diana Madden, Norah Crossnohere, Nicola Campoamor, John F. P. Bridges, Amy L. McGuire

**Affiliations:** 1 Center for Medical Ethics and Health Policy, Baylor College of Medicine, Houston, Texas, United States of America; 2 Department of Bioethics and Humanities, University of Washington School of Medicine, Seattle, Washington, United States of America; 3 Mary Ann & J. Milburn Smith Child Health Outcomes, Research, and Evaluation Center, Ann & Robert H. Lurie Children’s Hospital of Chicago, Chicago, Illinois, United States of America; 4 Department of Pediatrics, Feinberg School of Medicine, Northwestern University, Chicago, Illinois, United States of America; 5 Department of Internal Medicine, College of Medicine, The Ohio State University Wexner Medical Center; 6 Department of Biomedical Informatics, College of Medicine, The Ohio State University Wexner Medical Center, Columbus, Ohio, United States of America; Stanford University, UNITED STATES OF AMERICA

## Abstract

A technique known as investigative genetic genealogy (IGG) was first introduced to criminal investigations in 2018, and it has since been used by U.S. law enforcement to help identify hundreds of criminal perpetrators and unidentified human remains. As expertise in IGG grows, policymakers have shown interest in regulating it. To help inform these efforts and to promote coherence in IGG governance as it expands, we recruited experts representing a spectrum of IGG-relevant professions and perspectives to identify and prioritize IGG practices for policy attention and to develop policy options for addressing them. In two rounds of a modified policy Delphi, 31 participants prioritized nine IGG practices for policy attention. These top priority practices relate to: consent and notification; case eligibility and criteria; data management, privacy, and security; and governance and accountability. Participants expressed a range of opinions, some strongly held, and did not reach complete consensus with respect to any of the practices. However, convergence was strongest with respect to law enforcement participation in direct-to-consumer genetic genealogy databases against terms of service, which a large majority opposed and almost half evaluated as top priority for policy attention. Participants also voiced strong and consistent concern about management of data and samples collected and generated during IGG and the governance of private laboratories involved in IGG. Our study demonstrates the feasibility and value of engaging with diverse experts over an extended period on a pressing matter of public policy and provides a needed empirical foundation for IGG policymaking.

## Author summary

Since 2018, U.S. law enforcement has used investigative genetic genealogy (IGG) to help identify hundreds of violent criminals and unidentified human remains. The technique has raised privacy concerns, and various governmental and private actors are working to address these concerns through new laws, policies, and professional guidance. To help inform these efforts, we recruited diverse experts to deliberate on ethical, legal, and social issues relevant to IGG in a modified policy Delphi. Our study participants identified 33 IGG practices that one or more of them considered problematic, and they prioritized nine of those practices for policy attention. The nine practices relate generally to the cases that IGG is used to investigate, the processes by which direct-to-consumer genetic genealogy database customers and their relatives become involved in IGG, and the mechanisms in place to ensure that IGG is practiced effectively and responsibly by law enforcement, laboratories, and genetic genealogists. Our participants did not completely agree on any of the practices, although most opposed law enforcement participation in databases against terms of service and were concerned about how IGG data and samples are managed. Our study results are useful to those developing policies for IGG.

## I. Introduction

In 2018, investigative genetic genealogy (IGG) was first introduced to criminal investigations, and U.S. law enforcement has since used the technique to help identify hundreds of criminal perpetrators and unidentified decedents [1,2,3]. IGG involves uploading a genetic profile comprising single nucleotide polymorphisms (SNPs) from an unknown person (possibly collected from an item of evidence) to a direct-to-consumer (DTC) genetic genealogy database; building the person’s family tree from information about their genetic relatives identified by the database among database participants and public records; and developing leads as to the person’s identity from the family tree and case information [4,5,6]. FamilyTreeDNA (FTDNA) and GEDmatch are currently the only DTC genetic genealogy databases that permit IGG. Both have features that allow database participants to opt in to relative matching for specific IGG applications when conducted by self-identified members of law enforcement (known as law enforcement matching) [7,8,9].

IGG is the focus of a rapidly expanding media catalog including articles, books, podcasts, documentaries, and television series that detail its success in specific cases. As a result of this attention, the general public is becoming familiar with IGG, and studies have found broad support for its use for many investigative purposes [10,11,12]. Yet, from the beginning, IGG practice has been beset by ethical and legal questions related to, among other things, database terms of service, participant consent, and case eligibility criteria. Reflecting some of these concerns, as well as general distrust of the police, participants in focus groups sampled from four U.S. metro areas endorsed regulation of IGG [11].

Consistent with these public preferences, federal and state policymakers have begun to regulate the technique. In 2019, the U.S. Department of Justice adopted an interim policy for IGG conducted with federal funds or assistance (the DOJ Interim Policy) that restricts the kinds of cases eligible for IGG as well as the collection and use of DNA from non-suspects [13]. Since then, two states—Maryland and Utah—have passed comprehensive laws that also place limits on IGG [14,15], and Montana passed a succinct law that bans law enforcement from obtaining “familial DNA search results”from a consumer genetic database without a warrant [16]. More recently, some states have passed genetic privacy statutes—similar in many respects but not identical—that may limit IGG’s practice in their respective jurisdictions [17]. Complementing these efforts, new professional bodies are developing standards for IGG programs and practitioners to promote technical competence and ethical practice [18,19].

In sum, there is emerging support for regulating IGG in the United States that has translated into an increasing number of guidelines, laws, and other policies. To help inform these efforts and to promote coherence in the system of IGG governance as it expands, we convened an expert group to identify and prioritize IGG practices for policy attention and to develop effective and feasible policy options. This research is one component of The ForenSeq Study, a four-year project funded by the National Institutes of Health to study public preferences and policy options for IGG.

## II. Methods

### A. Ethics statement

The study protocol was reviewed and approved by the Institutional Review Board (IRB) of Baylor College of Medicine (H-47654).

### B. Study design

This research was designed as a modified policy Delphi. The Delphi method was developed by the RAND Corporation as a process of systematic group communication to facilitate technical forecasting [20,21]. A traditional Delphi usually involves the completion of a series of asynchronous questionnaires by experts whose identities are unknown to each other [20,21]. Its goal is to achieve consensus among these experts, which is treated as evidence that can inform decision-making processes [22]. By contrast, a *policy* Delphi facilitates deliberation of a major policy issue through highly structured, iterative rounds of engagement during which different positions on the issue and supporting evidence are exposed [23]. The goal of a policy Delphi is to identify a range of policy options and reasons for supporting or opposing each option [24]. In a *modified* policy Delphi, participants’ identities are known to each other and the process includes opportunities to react to evidence and assess various perspectives during in-person or virtual sessions [20,25].

**Fig 1:**
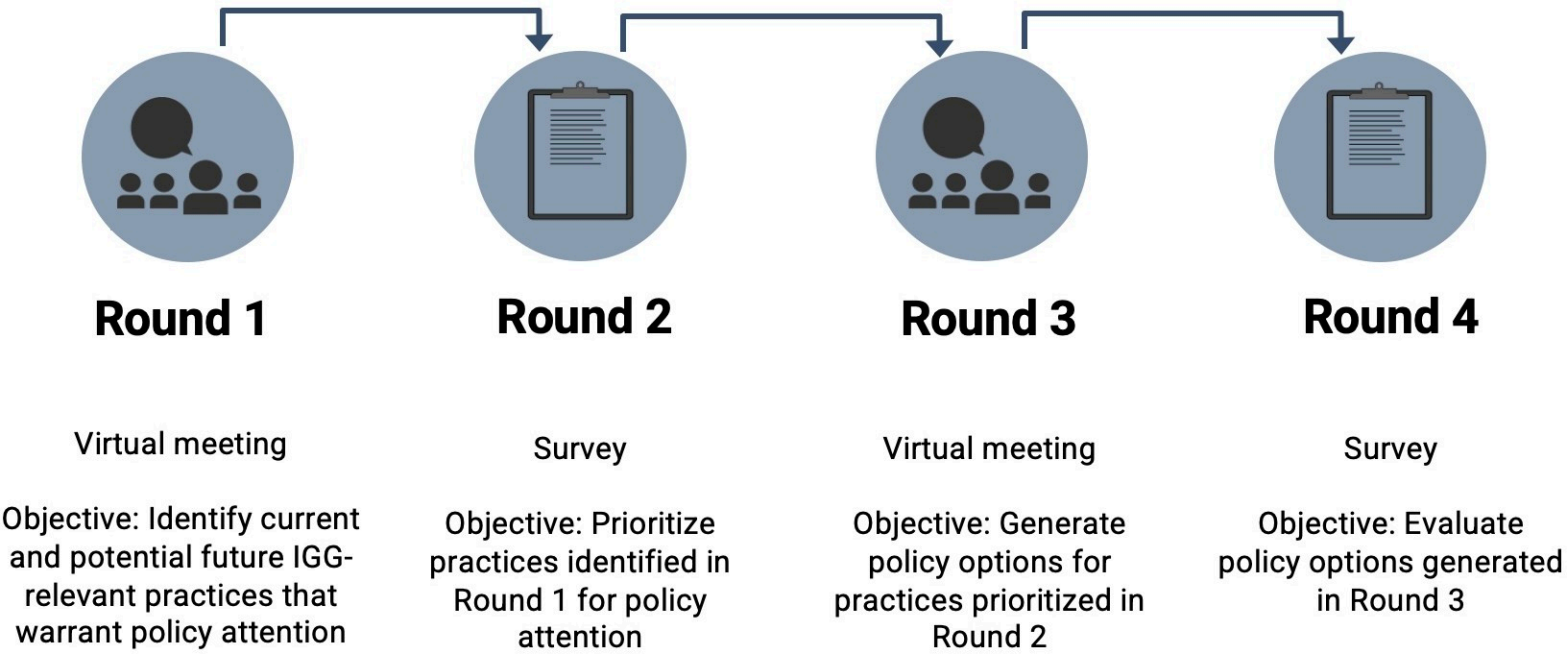
The ForenSeq Study modified policy Delphi.

We engaged participants in our modified policy Delphi in four iterative rounds that included two virtual meetings and two surveys (Fig 1). Here, we report the results of the first two rounds.

### C. Panel selection and recruitment

Delphi participants were selected based on their expertise in IGG and/or related fields, including genetic privacy and victim advocacy, to promote representation of a broad range of experiences and perspectives as well as demographic diversity. Expertise was determined from a review of professional activities, the peer-reviewed and gray literature, and media reports. In addition, a project consultant who is an expert in IGG suggested potential participants, although recruitment decisions were made by the project team. To be included, participants were required to be at least 18 years of age, able to participate in an English-language study, and reside in the United States, consistent with the focus of this research on U.S. policy.

One author contacted each candidate via email with an invitation that described the study’s purpose and the planned Delphi activities. Up to two follow-up emails were sent if the candidate did not respond to the initial invitation. No additional emails were sent after the second follow-up email. Most participants were recruited prior to the first round; four participants were recruited between the first and second rounds to increase diversity of perspectives based on the project team’s assessment.

### D. Data collection and analysis

Round 1. The first round was a virtual meeting that took place in April 2023. The meeting comprised facilitated discussions to identify ethical, legal, and social issues related to IGG that warrant policy attention. Facilitated discussions were organized according to four conceptual domains—consent; case selection; privacy and security; and governance—whose selection was guided by a literature review and related research activities [11,26]. In advance of the meeting, participants ranked each domain according to personal interest and/or knowledge to facilitate their assignment in meeting breakout groups.

The meeting began with introductions and background information about The ForenSeq Study, including preliminary results of related research activities. Information was also provided about the Delphi method and the specific processes in which participants would engage. Each participant then joined two successive breakout groups for facilitated discussion, where each group focused on one of the four conceptual domains and group assignment was based on earlier-solicited preferences. Each breakout group comprised 6–7 participants. The meeting concluded with a discussion session involving the full panel, during which representatives from each group reported key points from their breakout group’s discussion and all participants were given an opportunity to respond. Participants were offered $250 for participating in this round.

The entire meeting, including breakout group and full panel discussions, was video recorded; designated members of the project team also took detailed notes. After the meeting, the project team reviewed the recordings and notes for each session and prepared additional notes based on analysis of the recordings. In an iterative process that included characterizing specific points of concern and adjusting their scope to exclude overlap and to promote clarity and fidelity to the underlying data, the project team developed an initial list of 37 current and potential future IGG practices that, according to one or more participants, warrant policy attention. These items were framed as practices, rather than ethical, legal, or social issues, consistent with the round 1 discussions and to facilitate the development of policy options in later Delphi rounds. Four practices were removed from the initial list based on team consensus that the quantity and/or quality of discussion about them did not support adequate characterization or further consideration by the full panel.

For each of the remaining practices, a brief description was drafted and refined during multiple rounds of review and discussion among the project team. The conceptual domain labels were then adjusted to better align with round 1 discussions, and the 33 practices were organized according to the revised domains.

Round 2. We developed a 45-minute online survey that asked panelists to prioritize the 33 practices identified in round 1 for policy attention. The survey is reproduced in the online Supplement (S1 Survey). The first section described the survey and the potential risks and benefits of participation. Informed consent was provided by clicking on the button to proceed with the survey; the IRB approved a waiver of written documentation of informed consent. The second section provided definitions of terms used in the survey, including “policy” and “practice,” as well as an overview of the practice domains and survey flow.

The third section was organized by conceptual domain. Consistent with best practices for accuracy testing [27], each domain was introduced with a background statement intended to capture critical discussion points raised during round 1. Participants were then asked to evaluate each of the IGG practices in the domain using a four-point Likert-type scale with the following response options: “high priority,” “medium priority,” “low priority,” and “no priority.” An “unable to prioritize” response option was included, although per policy Delphi best practices, participants were encouraged to use it sparingly [24] and also were provided an opportunity to explain this selection when made. At the end of each domain, participants were invited to respond to the following open-ended question: “Is there anything you would like to share about [domain]?” Responding to this question was made optional to reduce burden on participants. After participants completed their evaluation of practices in all domains, they were shown the practices they had selected as “high priority” in any domain and asked to select up to three practices as “highest priority.”

The fourth section of the survey included questions asking whether panelists favored or opposed specific practices using a five-point Likert-type scale with the following response options: “strongly oppose,” “somewhat oppose,” “neutral,” “somewhat favor,” and “strongly favor.” The survey concluded with the following optional, open-ended question: “Is there anything you would like to share about the practices you most strongly oppose, most strongly favor, and/or you believe warrant the highest priority for policy attention?”

The survey was programmed using the online survey platform Qualtrics. Delphi participants were emailed the link to the survey and sent up to three email reminders to complete it. Upon completion, they were offered $100 for participating in this round. Surveys were administered in August and September 2023.

We used Excel to generate counts, frequencies, and scores for policy prioritization of each IGG practice. A “general priority score” was calculated by summing counts of responses multiplied by a value for each item as follows: (“high priority” count * 4) + (“medium priority” count * 3) + (“low priority” count * 2) + (“no priority” count * 1) = general priority score, with a maximum total possible general priority score equal to 124, where the higher score means higher priority. “Unable to prioritize” counts were not included in the general priority score. To account for these responses, we also calculated an “average priority score” as follows: ((“high priority” count * 4) + (“medium priority” count * 3) + (“low priority” count * 2) + (“no priority” count * 1)) / (31 –“unable to prioritize” count) = average priority score, with a maximum total possible average priority score equal to 4. The project team agreed on four selection criteria for identifying “top priority” IGG practices: general priority score ≥85; average priority score ≥3; “highest priority” count ≥4; and “high priority” count ≥10. Multiple criteria were selected to account for subtle variations in rankings that appeared across scores and counts, increasing confidence in our determination of convergence. Cut-off scores and counts were selected based on our visual inspection of data distributions. Finally, we reviewed responses to optional, open-ended questions, including participants’ reasons for their rating selections and inability to prioritize specific practices. Given the limited number of open-ended comments, coding of these data was deemed unnecessary.

## III. Results

### A. Participants

A total of 44 individuals were invited to participate in the Delphi and 34 accepted, for a response rate of 77%. Of those who did not accept, four did not respond to emailed invitations and six either did not receive employer approval or declined to participate due to time constraints or other issues. The round 1 virtual meeting was attended by 27 participants; the round 2 survey was completed by 31 participants. Three did not participate in either round.

Almost half of recruited participants were age 50 and over (n = 16) and exactly half identified as female (n = 17) (Table 1). Also, most participants identified as non-Hispanic White (n = 26) and resided in the U.S. South or West (n = 22). Participants were asked to select among seven IGG-relevant professional areas as their primary or secondary area of expertise: law enforcement, forensic science, genetic genealogy, database operations, legal practice, academic law or ethics, and advocacy. Of the 32 participants who provided this information, exactly half (n = 16) selected only a primary area of expertise. Twelve unique combinations of the seven areas of expertise were represented by the remaining participants (n = 16) who selected both primary and secondary areas of expertise. The most frequently selected primary or secondary area was genetic genealogy (n = 13), followed by law enforcement (n = 10); the majority of those who selected one of these areas also selected a second area of expertise.

**Table 1:** Recruited Delphi participant characteristics (N = 34).

Characteristic	n (%)^a^
Age, in years	
≤39	5 (15)
40–49	10 (29)
≥50	16 (47)
Unknown/prefer not to answer	3 (9)
Gender	
Female	17 (50)
Male	14 (41)
Other/unknown/prefer not to answer	3 (9)
Race and ethnicity^b^	
Non-Hispanic White	26 (76)
Other	3 (9)
Unknown/prefer not to answer	5 (15)
Geographic location^c^	
U.S. Northeast	4 (12)
U.S. Midwest	5 (15)
U.S. South	10 (29)
U.S. West	12 (35)
Unknown/prefer not to answer	3 (9)
Expertise^d^	
Academic law or ethics	6 (18)
Advocacy	5 (15)
Database operations	3 (9)
Forensic science	5 (15)
Genetic genealogist	13 (38)
Law enforcement	10 (29)
Legal practice	6 (18)
Unknown/prefer not to answer	2 (6)

^a^ Sum of percentages may not equal 100% due to rounding.

^b^ Respondents were asked to select “all that apply”; these data are reported as mutually exclusive. “Other” includes respondents who selected one or more of the following categories: Indigenous Peoples; Asian; Black or African American; Hispanic or Latino; Middle Eastern or North African; Native Hawaiian or other Pacific Islander; or self-describe.

^c^ As defined by the U.S. Census Bureau.

^d^ Not mutually exclusive. The number for each domain indicates the number of participants who selected it as their primary or secondary area of expertise. Sixteen participants selected only a primary area of expertise; sixteen participants selected both a primary and secondary area of expertise; and two participants did not select an area of expertise.

### B. Round 1

Round 1 participants identified 33 current or potential future IGG practices that warrant policy attention (Table 2). The practices were organized by four conceptual domains: consent and notification; case eligibility and selection; data management, privacy, and security; and governance and accountability. There were 7–10 practices per domain.

**Table 2:** IGG practices identified for policy attention in round 1.

Conceptual domain^a^	Practice, short label	Practice, full descriptive statement
Consent and notification	Different database consent approaches	Databases have adopted different default consent approaches to IGG: one opts U.S. database participants in to IGG and the other opts all participants out.
	No specific consent	Databases do not obtain specific consent to each instance of IGG, but rather they obtain broad consent to IGG in all cases.
	Complex consent forms	Database consent forms can be long and complex, which might interfere with database participants’ comprehension.
	Database policy changes	Databases can change their policies and terms of service at any time.
	*Law enforcement disregard of consent selections*	*Law enforcement is generally allowed to participate in databases against terms of service and to disregard the consent selections of database participants when they conduct IGG*.
	No notification to database participants	Databases currently do not notify, or offer to notify, database participants when they are identified as a genetic relative of the unknown DNA sample.
	No consent from genetic relative not participating in databases	Databases do not currently obtain consent to IGG, or require participants to obtain consent to IGG, from genetic relatives of database participants who are not themselves database participants.
	Surreptitious collection of reference samples	Law enforcement is generally allowed to collect reference samples surreptitiously and without consent from reference testers.
Case eligibility and selection	Different database eligibility criteria	Each database has identified a different set of IGG-eligible crimes.
	Different state crime definitions	States have adopted different definitions for IGG-eligible crimes.
	IGG restriction to violent crimes	Database rules and other policies currently restrict IGG to violent crimes and do not permit its use for non-violent crimes.
	No exceptions to eligibility criteria	Database rules and other policies do not currently allow any exceptions to case eligibility criteria.
	*IGG underuse for defense/ exoneration purposes*	*IGG is used by defense and post-conviction attorneys for the purpose of exonerating persons wrongly accused of or convicted for crimes less frequently than by law enforcement and prosecutors*.
	IGG underuse for victims from marginalized communities	IGG is used to investigate cases involving victims from marginalized communities less frequently than those involving other victims, especially victims of European descent.
	IGG use in Baby Doe cases	Use of IGG to identify Baby Does has led to prosecution of mothers that might be unjustified or disproportionate.
	IGG use in active investigations	Law enforcement can generally use IGG in investigations of active cases and does not need to wait until all other techniques have been attempted and failed and cases have gone cold.
	Inconsistent/underuse of cost-benefit analysis	Law enforcement might not sufficiently or consistently take into account the costs of IGG, relative to the likelihood of its success, when deciding whether to use IGG in specific cases.
	*IGG without direct STR comparison*	*It is possible to conduct IGG even when a direct comparison of STR profiles to confirm identity is not possible*.
Data management, privacy, and security	*Inconsistent/inadequate data and sample management*	*Practices for recording*, *storing*, *and transferring DNA samples and related data collected and generated during IGG are inconsistent and might not be sufficiently protective of individual privacy*.
	Inconsistent/inadequate data preservation	Practices for preserving information developed during IGG are inconsistent and might undermine individual privacy.
	Security vulnerabilities of databases	Genetic genealogy databases might not be sufficiently secure to prevent unauthorized access to or disclosure of information in the database.
	Law enforcement use of medical data	Law enforcement might use medical information from SNP profiles developed during IGG in their investigations.
	*IGG in medical/research databases*	*Law enforcement might conduct IGG in medical and research databases*.
	*Development of unregulated databases*	*Law enforcement might use data from DNA samples collected during IGG to populate unregulated law enforcement databases*.
	*Laboratory reuse of data and samples*	*Laboratories that develop SNP profiles for IGG might reuse DNA samples and related data for other purposes*.
Governance and accountability	*Patchwork governance of IGG*	*IGG currently is governed by a patchwork of laws*, *guidelines*, *policies*, *and best practices*, *rather than a standardized governance framework*.
	Self-regulation of IGG	In some jurisdictions, IGG is governed largely by guidelines, best practices, and other self-regulatory mechanisms developed for IGG by expert groups.
	No judicial oversight of IGG	Judicial oversight of IGG is not required in most jurisdictions.
	Insufficient penalties for non-compliance	Some governance mechanisms might not include sufficient penalties for noncompliance.
	Limited remedies for persons harmed by IGG	There are limited remedies available to those who are harmed by IGG.
	Limited oversight of genetic genealogists	Genetic genealogists are generally not subject to governance mechanisms that promote quality, such as licensing requirements.
	*Limited quality oversight of private laboratories*	*Private laboratories are subject to few governance mechanisms that promote SNP profile quality*, *such as requirements regarding use of validated techniques*.
	Limited transparency about IGG uses/outcomes	Information about IGG uses and outcomes is generally not collected and shared with the public.

IGG = investigative genetic genealogy; STR = short tandem repeat; SNP = single nucleotide polymorphism; Baby Doe = unidentified decedent who is a newborn or infant; reference tester = a genetic relative of a person whose identity is under investigation from whom DNA is collected and tested to help narrow the scope of the investigation by law enforcement; reference sample = DNA collected from a reference tester.

^a^ Conceptual domains and full descriptive statements were provided in the round 2 survey. Short labels were not provided in the round 2 survey. Italicized practices were prioritized for policy attention in round 2.

### C. Round 2

Round 2 participants prioritized the 33 IGG practices for policy attention. Nine practices satisfied all selection criteria for top priority (Table 3). Evaluation data for practices that did not meet the top priority criteria are set forth in the Supplement (S1 Table). In responses to optional, open-ended questions quoted below, participants are referenced by their assigned study ID.

**Table 3:** IGG practices evaluated as top priority in round 2 (N = 31).

Practice, short label^a^	Criteria used for prioritization				Other counts (not used for prioritization)			
	General priority score	Avg priority score	Highest priority count	High priority count	Medium priority count	Low priority count	No priority count	Unable to prioritize count
Law enforcement disregard of consent selections	99	3.5	15	21	3	2	2	3
IGG in medical/research databases	96	3.2	5	16	6	6	2	1
Patchwork governance of IGG	98	3.3	6	15	8	7	0	1
Limited quality oversight of private laboratories	104	3.5	7	19	7	3	1	1
Laboratory reuse of data and samples	107	3.5	4	18	10	2	1	0
Inconsistent/inadequate data and sample management	106	3.5	4	18	11	0	1	1
Development of unregulated databases	97	3.1	4	17	3	9	2	0
IGG underuse for defense/exoneration purposes	89	3.3	5	16	5	4	2	4
IGG without direct STR comparison	89	3.1	6	13	9	3	4	2

^a^ See Table 2 for each practice’s full descriptive statement.

#### Consent and notification domain

Only one IGG practice in the consent and notification domain met all top priority criteria: law enforcement’s potential disregard of database participants’ consent selections—also known as database participation “against terms of service.” Notably, this practice was selected as “highest priority” by more participants (n = 15) than any other practice in the dataset and by more than twice the number of participants who selected the practice with the second largest “highest priority” count. In response to the favor-oppose question tied to this practice (Table 4), two participants strongly favored it, while 27 participants somewhat or strongly opposed it. Representing this majority view, one participant remarked that “the single most important issue is LE [law enforcement] violating terms of service to upload to MyHeritage,” a DTC genetic genealogy database that does not permit IGG (030). However, a different participant who selected “unable to answer” stated that they “would like more information on how and when this happens. and how this is or isn’t differ[e]nt from other LE investigation techniques” (019).

**Table 4:** Favor-oppose questions evaluated in round 2 (N = 31).

Practice	“In this last section, we will ask if you oppose or favor certain practices. What is your view of this practice?” n (%)^a^				
	Strongly favor	Somewhat favor	Neutral	Somewhat oppose	Strongly oppose
Use of IGG to investigate non-violent crimes	2 (6)	6 (19)	4 (13)	7 (23)	12 (39)
Use of IGG to investigate active cases before all other techniques have been attempted and failed and the cases have gone cold	14 (45)	4 (13)	3 (10)	5 (16)	5 (16)
Use of IGG to identify Baby Does	8 (26)	13 (42)	2 (6)	6 (19)	2 (6)
Use of IGG when an STR profile cannot be developed from the unknown DNA sample and so cannot be used to confirm identity	10 (32)	3 (10)	8 (26)	6 (19)	4 (13)
Default consent approach to IGG that opts all participants in to IGG	6 (19)	7 (23)	4 (13)	6 (19)	8 (26)
Default approach of obtaining consent to all eligible uses of IGG rather than consent to each specific use	15 (48)	5 (16)	5 (16)	2 (6)	4 (13)
Law enforcement participation in databases against terms of service	2 (6)	0 (0)	2 (6)	7 (23)	20 (65)
Notification to database participants when they are identified as a genetic relative of an unknown DNA sample during IGG	1 (3)	6 (19)	5 (16)	8 (26)	11 (35)
Surreptitious collection/testing of DNA from suspects to confirm their identity	19 (61)	5 (16)	2 (6)	2 (6)	3 (10)
Surreptitious collection/testing of DNA from genetic relatives who are not suspects to help focus an investigation	3 (10)	5 (16)	1 (3)	7 (23)	15 (48)
Laboratory reuse of DNA samples collected and tested during IGG for research purposes	0 (0)	1 (3)	5 (16)	6 (19)	19 (61)
Use of IGG only with specific permission from a court and subject to its oversight	6 (19)	4 (13)	2 (6)	8 (26)	11 (35)
Mandatory licensure of genetic genealogists who conduct/facilitate IGG	12 (39)	10 (32)	3 (10)	2 (6)	4 (13)
Regulation of IGG via federal law	8 (26)	11 (35)	6 (19)	2 (6)	4 (13)
Regulation of IGG via individual state laws	9 (29)	10 (32)	5 (16)	4 (13)	3 (10)
Regulation of IGG via best practices and guidelines developed by IGG practitioners/stakeholders	8 (26)	13 (42)	4 (13)	2 (6)	4 (13)

^a^ Sum of percentages may not equal 100% due to rounding. Shading corresponds to selection of combined “somewhat favor” and “strongly favor” response options, or combined “somewhat oppose” and “strongly oppose” response options, by at least 60% of respondents (N = 31), with darker shading corresponding to a larger proportion of respondents, at 10% increments.

Many of the other practices in this domain landed at the bottom of the dataset with respect to prioritization. For example, the failure of databases to notify (or offer to notify) database participants who opted in to IGG when they are identified as a genetic relative of a forensic unknown was evaluated as “low priority” or “no priority” by more participants (n = 21) than any other practice in the dataset. In a related favor-oppose question, most participants somewhat or strongly opposed such notifications (n = 19).

That there is no requirement to obtain consent to IGG from genetic relatives of database participants also scored low on all top priority criteria. Indeed, it received the lowest general priority score, lowest average priority score, and highest “no priority” count (n = 13) of all the practices in the dataset. Open-ended responses suggest reasons for this finding. One participant characterized a requirement that databases or database participants obtain consent from genetic relatives before opting in to law enforcement matching as an “impossible and unreasonable ask” (010); another echoed the opinion that such a requirement would be “impossible to execute” (001).

By contrast, the practice of collecting surreptitious DNA samples from non-suspects did not meet all top priority criteria, although most participants somewhat or strongly opposed this practice (n = 22). In open-ended responses, one participant suggested that relevant policies need clarification; another urged a blanket prohibition on this practice. A different participant shared their personal observation that surreptitious DNA collections are “extremely uncommon primarily because it is super expensive. It is [a] lot easier to simpl[y] find a person willing to consent” (001), but it is unknown whether this was a common experience among participants.

#### Case eligibility and selection domain

The case eligibility and selection domain included the largest number of practices for consideration, but only two of them were evaluated as top priority: perceived underuse of IGG for defense and exoneration purposes and its potential use even when direct comparison of STR profiles to confirm the identity of an IGG-generated lead is not possible. The first practice—IGG’s underuse for defense and exoneration purposes—was selected as “unable to prioritize” by four participants, who noted potential practical issues for defense and post-conviction attorneys related to DNA sample access, testing motivation, and timing that might be difficult to completely resolve through policy. Notwithstanding these barriers, one participant remarked that, at the very least, “IGG as a tool for exoneration should be more publicized” (010).

The second practice—proceeding with IGG even when lead identity cannot be confirmed via direct comparison of the lead’s STR profile with the forensic STR profile—was considered a non-starter by one participant: “If there is not a good STR for confirm[ing] the identity we should not be doing IGG. Super simple rule” (002). However, a different participant cautioned that such judgments might be premature, opining that “the science and the legal side need to be worked through before policy should be discussed” (041). Perhaps reflecting this uncertainty, while thirteen participants somewhat or strongly favored proceeding with IGG even when an STR profile cannot be developed from the forensic sample, this favor-oppose question had the largest number of neutral responses (n = 8). Importantly, a participant’s support for this practice did not mean the participant rejected other methods of post-IGG identity confirmation, such as direct comparison of SNP profiles.

Responses to open-ended items and other questions related to this domain trended towards non-intervention in case eligibility policies at this time. That is, most participants agreed that IGG should include investigation of active cases before they have gone cold (n = 18) but should not be extended to non-violent crimes (n = 19), consistent with current policies. Still, in open-ended comments, three participants supported relaxing case eligibility criteria to permit IGG to investigate crimes that are “egregious” or threaten “imminent harm” (012, 024) but technically might be classified as non-violent, such as the manufacture of improvised explosive devices. A different participant advocated against this approach, however, warning that “strict criteria” are needed to avoid a “very serious slippery slope” toward inappropriate or lower priority applications of IGG (030).

With respect to using IGG to identify deceased newborns and infants (known as Baby Doe cases), the response to a related favor-oppose question and open-ended comments were generally supportive. Twenty-one participants somewhat or strongly favored using IGG to investigate these cases, while eight opposed it. In comments, several participants voiced strong support for Baby Doe applications, with some taking issue with the survey’s connection of Baby Doe investigations with the potentially unjustified or disproportionate prosecution of mothers. For example, one participant stated: “This is not an IGG issue. All living human beings have the right to be identified. The issue isn’t whether we should investigate these Crimes[,] the issue is how we should prosecute the crimes” (034).

#### Data management, privacy, and security domain

The data management, privacy, and security domain included the smallest overall number of practices for consideration and received few open-ended responses. Yet the practices in this domain are notable for their consistently high rankings across many criteria. Indeed, four of the nine top priority practices, as well as four of the five practices in the dataset that received a general priority score over 100, were in this domain.

The first top priority practice is laboratories’ potential reuse of SNP profiles for purposes unrelated to case investigations, such as for commercial or private research purposes. This practice received a general priority score of 107—the highest in the entire dataset—and reuse for research purposes was somewhat or strongly opposed by 25 participants in a related favor-oppose question. The second top priority practice is inconsistent and potentially privacy-inadequate management of DNA samples and data collected or generated during IGG, which received a general priority score of 106. Although one participant indicated that FTDNA and GEDmatch have relevant protocols, a different participant saw potential positive consequences of some kind of policy intervention to promote privacy and security. Focusing on consequences for the courtroom, they remarked that “clear policy. would increase the utility of IGG in criminal investigations because the current lack of regulation causes many motions to dismiss/exclude due to the possibilities of what could have been done with the information” (033).

The third top priority practice in this domain is law enforcement’s potential population of unregulated databases with SNP profiles, but evaluations of this practice were more mixed compared to other top priority practices. Although it satisfied all top priority selection criteria, about one-third of participants (n = 11) still evaluated it as “low priority” or “no priority.” The last top priority practice is the potential conduct of IGG in medical and research databases. In the only open-ended response related to this practice, a participant asked whether there exist any medical or research databases that could be a site for IGG. This comment might have been alluding to the fact that IGG requires a large-scale SNP database with an internal algorithm that compares profiles and generates kinship predictions.

#### Governance and accountability domain

Finally, two practices in the governance and accountability domain were selected as top priority. First, respondents generally agreed that IGG’s current governance by a patchwork set of laws, guidelines, policies, and best practices, rather than a standardized framework, requires policy attention. Notably, this was the only top priority practice to receive zero “no priority” evaluations. In an open-ended response, however, one participant questioned whether harmonizing requirements across jurisdictions—although “desirable”—is feasible given the sheer number of jurisdictions with authority over IGG and its practitioners (008). In related favor-oppose questions, participants did not express a clear preference for who should regulate IGG. More than half somewhat or strongly favored regulation via federal law (n = 19), individual state laws (n = 19), and/or best practices and guidelines developed by IGG practitioner/stakeholder groups (n = 21).

Second, participants supported policy attention to governance of commercial laboratories—in particular, their activities related to generating SNP profiles for IGG. Under-regulation of these activities was selected as “highest priority” (n = 7) and “high priority” (n = 19) by more participants than any other practice after database participation against terms of service. Although it was noted that some private laboratories supporting IGG are accredited, one participant believed that “it is important that all labs doing such work be accredited” to ensure accuracy of the data they generate (012). Consistent with this opinion, another participant questioned the logic of not subjecting laboratories that generate SNP profiles for IGG to the same standards as laboratories that generate STR profiles for CODIS.

With respect to other practices in this domain, the absence of judicial oversight of IGG in most jurisdictions was not evaluated as top priority. Although one participant described this kind of oversight as “crucial” (031), in a related favor-oppose question, it was somewhat or strongly opposed by most participants (n = 19). By contrast, limited legal remedies available to those harmed by IGG was one of only three practices in the entire dataset to receive zero “no priority” evaluations. However, it did not meet all top priority criteria, with several participants seeking clarification as to who and how individuals might be harmed by IGG. Representative of these comments, one participant questioned the assumption that IGG harms “genetic witnesses,” or non-suspects identified as genetic relatives of perpetrators: “I would need to hear more about who is claiming to have been harmed by IGG simply by being related to a suspect. Typically a genetic witness’s information should never be disclosed publicly” (024).

Finally, the general absence of licensure requirements for genetic genealogists, or other governance mechanisms to promote the quality of their work, was not rated as top priority, yet most participants (n = 22) somewhat or strongly favored mandatory licensure. For example, one participant argued that “[i]f they are going to provide investigative lead results to law enforcement, [IGG practitioners] should welcome the scrutiny of their work and be accepting of some sort of certification” (005). But another participant remarked that licensure “seems unnecessary” given “that the results of IGG are not themselves the basis for an arrest warrant” (012). Representing yet a different view, a third participant argued for limiting the practice of IGG to law enforcement, which presumably would moot the licensure debate: “The genetic genealogy portion of IGG *IS* police work. It should only be done by police. Only law enforcement officers have the training, oversight, and have signed up to take the responsibility for an investigation working or not working” (009) They concluded: “Train cops to do genealogy or train genealogists to be cops.” (009)

## IV. Discussion

Consistent with the design of our research process as a modified policy Delphi, participants voiced diverse opinions, some strongly held, as to which IGG practices warrant policy attention and how to prioritize them. Indeed, complete consensus was not reached with respect to any practice. Nevertheless, through structured, evidence-informed group discussions followed by independent, asynchronous evaluations, participants surfaced important points of consideration with respect to almost three dozen IGG practices and converged on prioritization of nine of them.

Of the three IGG practices that received zero “no priority” evaluations, only one—IGG’s patchwork governance—met all top priority criteria. This finding indicates that the proliferation of laws, regulations, and standards for IGG might be cause for concern. Among other things, it creates confusion and distress for IGG practitioners about their obligations and the limits on their activities when they are subject to conflicting policies—some of which might include strict penalties for non-compliance. It is unclear, however, what issues would most benefit from policy harmonization or how best to achieve it. These questions are a focus of the last two rounds of the Delphi.

There was varying strength of support for prioritizing each of the nine top priority practices, with one major exception—law enforcement’s potential participation in DTC genetic genealogy databases against terms of service. Although two participants supported this practice, perhaps for reasons described in a different study [26], it was selected as “highest priority” for policy attention by considerably more participants than any other practice in the dataset and was somewhat or strongly opposed by a large majority of participants. Maryland and Utah address this issue by encouraging or requiring law enforcement compliance with database terms of service [14,15]. Scientists also have proposed technical solutions that include implementation of technologies and processes that would help databases detect and block attempted uploads of DNA profiles that were not developed by recognized DTC genetic testing providers [28,29].

Separately, warrant-based database participation is sanctioned in some recently enacted state genetic privacy laws [17], as well as the Montana IGG law, which prohibits law enforcement from obtaining what is described as “familial DNA search results” from a consumer DNA database without a warrant [16]. There are legal questions about whether warrants to conduct IGG can satisfy Fourth Amendment requirements that searches are particularized and supported by probable cause as well as legal questions about who has standing to challenge such warrants [30,31,32,33]. But if warrants to conduct IGG can survive constitutional challenges, the privacy benefits these laws hope to achieve should be examined. While requiring a warrant to conduct IGG might appear more privacy protective than the status quo, warrant-based participation in DTC genetic genealogy databases can be, depending on the terms of the warrant, the functional equivalent of participation against terms of service. In both cases, an entire database might be made available for law enforcement matching, regardless of the choices made by individual database participants to opt in or out of law enforcement matching [33]. In fact, in the one known instance of warrant-based participation in a genetic genealogy database [34], law enforcement requested information from GEDmatch for all participants identified by the database as genetic relatives of a criminal perpetrator who were opted out of law enforcement matching by the database following a policy change [35]. Warrant-based participation also could open the floodgates to law enforcement participation in larger databases than FTDNA and GEDmatch [36,37] that are currently off limits to law enforcement because they prohibit use for any law enforcement purpose and possibly also databases that do not accept uploads of DNA profiles developed by third parties. The practical consequences of a warrant requirement with respect to individual privacy might not be widely appreciated, but they merit attention in policy discussions.

Given that issues of participant consent and autonomy dominate legal and ethical commentary regarding IGG, it is notable that none of the other practices in the consent and notification domain were evaluated as top priority by our expert participants. Many of those practices are within the control of the databases and have evolved over time in the direction of providing greater transparency around law enforcement’s permitted activities and obtaining database participants’ affirmative consent to law enforcement matching. (Departing from the affirmative consent trend, in 2021 GEDmatch opted all participants in to law enforcement matching to help identify unidentified human remains [38].) Although there are still differences in database consent approaches, these differences were not prioritized for policy attention. Most participants also rejected replacing general consent to law enforcement matching, which is the current approach of both FTDNA and GEDmatch [7,8,9], with a requirement to obtain specific consent every time law enforcement conducts IGG, which may be required by some new state genetic privacy statutes [17].

Further, most participants rejected the hypothetical practice of obtaining consent to IGG from genetic relatives of database participants; the absence of a relational consent requirement received the lowest general priority score, lowest average priority score, and highest “no priority” count of all the practices in the dataset. One objection to IGG is that it intrudes on the privacy interests of individuals who are not database participants and have not consented to being identified on an investigative family tree but nevertheless appear on that tree by virtue of the unilateral actions of their database-participating relatives. Thus far, few have explored the possible implications of these objections for basic family research uses of DTC genetic genealogy databases. These uses include, for example, adoptees and donor-conceived individuals researching the identity of biological parents who might have received assurances that their identities would not be disclosed. These uses also include individuals researching their family tree who learn via genetic genealogy that they were conceived in circumstances (involving, for example, an affair or sexual assault) that some relatives prefer to keep secret. An increasing number of studies have found that family research uses of DTC genetic genealogy databases—while a positive and valuable experience for many—can lead to discoveries that have profound, long-lasting, and potentially devastating consequences for some database participants as well as their non-participating family members [39,40,41,42]. If the absence of non-participants’ consent to IGG is ethically problematic, is it also ethically problematic for family research using genetic genealogy tools more generally? What are the ethically relevant similarities and differences of these uses, and what do they imply for addressing the interests of non-participating family members in either context? We believe these questions are worth exploring, although this did not occur during the Delphi. Instead, expert comments focused on the difficulty of operationalizing a relational consent requirement. As others have noted, given that a database will identify hundreds or thousands of a participant’s genetic relatives, many of whom were likely not even known to the participant until after the database identified them, it would seem impossible to satisfy such a requirement for reasons of both numerosity and timing [43]. In the end, although some issues of individual consent might have risen to the top of Delphi participants’ list of concerns a few years ago, in mid-2023, most were judged to be low priority.

The majority of participants also believed that current case eligibility criteria set forth in the DOJ Interim Policy, Maryland and Utah laws, and FTDNA and GEDmatch terms of service are generally acceptable, or at least tolerable, at this time. According to these criteria, IGG may be used to investigate active cases and Baby Doe cases but generally not non-violent crimes [7,8,9,13,14,15]. However, some participants favored relaxing strict eligibility criteria to make room for exceptional cases. A reasonable objection to permitting exceptions is that they might become common if standards for granting them are vague or non-existent. Requiring a court, committee, or multiple agency officials to review and approve exceptions—similar to the oversight mechanisms built into the Utah and Maryland laws [14,15]—could help to avoid this outcome.

Otherwise, participants evaluated only one case-related practice as top priority: IGG’s perceived underuse by defense and post-conviction counsel. It is possible that the exceptionally small number of known defense uses of IGG has masked its true potential to help the wrongly accused and convicted. If so, one policy response might be IGG education and training programs for the defense bar. Funding also might be a significant issue. IGG is not free; unlike law enforcement agencies with in-house IGG capabilities, defendants often will need to contract with laboratory, bioinformatics, and/or genetic genealogy professionals to support the technique. Although some vendors might offer their services at reduced rates or pro bono, both FTDNA and GEDmatch charge for IGG-related uploads to their databases. Another possible issue is that database IGG policies are focused on law enforcement actors. More research is needed to understand facilitators and barriers to defense uses of IGG in order to craft effective policy solutions.

Finally, our participants converged on concern about the involvement of private laboratories in the development of SNP profiles for IGG: two of the nine prioritized practices concern laboratory activities, and they received some of the highest general priority scores in the study. Although accredited forensic laboratories are beginning to acquire specialized equipment to support IGG, many agencies continue to outsource work to private laboratories. This issue is already the focus of policy discussions. The policy and procedure subcommittee of the National Technology Validation and Implementation Collaborative (NTVIC) working group on IGG has identified accreditation status as an important consideration in agency evaluations of potential laboratory partners [18]. Furthermore, the Maryland IGG law requires a state agency to develop a licensure program for laboratories supporting IGG [14], although the deadline for establishing the program has passed without public action [44]. By contrast, the potential for private laboratories to reuse IGG data and samples for commercial purposes has not yet been a focus of policy discussions, perhaps because the commercial opportunities for reuse are not well-known and there have not been any reported instances of a private laboratory selling IGG data or samples.

This study is subject to limitations. First, only three participants selected database operations as a primary or secondary area of expertise. It is possible that our results related to evaluation of database practices would have been different if the panel had included more database representatives. To increase representation of their perspective, future recruitment strategies should prioritize past and current employees of DTC genetic service companies. Second, approximately two-thirds of participants resided in the U.S. West and South, likely reflecting law enforcement’s swifter uptake of IGG in these regions. It is unclear if this geographical imbalance impacted study results, but future research should attempt greater representation of U.S. Midwestern and Northeastern participants to address this concern.

Third, responses to open-ended items in the round 2 survey were optional; therefore, the reported responses were selected from a smaller sample than if responses had been required. Based on our review of round 1 discussion data, however, we believe they are an adequate representation of participants’ perspectives. Further, open-ended responses are likely biased in favor of strongly held opinions, although policymakers may appreciate being made aware of these opinions.

Fourth, several months passed between the round 1 engagement session and round 2 survey. Some participants might have responded to survey questions differently if they had been asked those questions soon after the engagement session, when opposing considerations and arguments were still fresh on their minds. To help address this issue, the survey reminded participants of points made during the engagement session via discussion summaries. Regardless, considerable time and resources are required to analyze data from a Delphi round and to develop a detailed survey from those findings. Given these constraints, we believe the four months that passed between rounds 1 and 2 was reasonable.

Finally, and also related to timing, the research activities took place in mid-2023, but the IGG policy and business landscape has changed since that time. Among other things, in November 2023, NTVIC expanded on and updated guidelines for crime laboratories and investigative agencies engaged in IGG-relevant activities [18]. The following month, a different professional body, the Investigative Genetic Genealogy Accreditation Board, released professional standards (later revised in 2024) and a code of ethics for genetic genealogists who conduct IGG [19,45]. In January 2024, Othram, a private laboratory, and Gene by Gene, which operates the FTDNA database, entered an agreement whereby Othram assumed responsibility for triaging and uploading IGG data to the FTDNA database [46]. More recently, Qiagen, which owns GEDmatch through its subsidiary Verogen, announced a partnership with Bode Technologies, a forensics laboratory company, according to which Bode will manage Verogen’s commercial transactions related to IGG [47]. On the same day, Othram and Qiagen announced a commitment to ensuring technological compatibility across platforms and exploring alignments around informed consent and case eligibility criteria [48]. It is possible that participants might evaluate some practices differently today given these and/or other developments.

## V. Conclusion

There is support for and increasing interest in regulating IGG to promote privacy. However, there is a danger that these efforts might be ineffectual or even harmful if they are not guided by accurate information about IGG’s processes, inputs, and outputs. This includes, at a minimum, accurate information about what law enforcement can (and cannot) learn about individuals and their families from IGG and what law enforcement can (and cannot) do with what they learn. Effective policies for IGG will be those designed to address specific issues grounded in facts related to its current practice and plausible predictions about its potential future practice. They also will be carefully crafted to maximize societal benefits and to avoid unintended negative consequences. What is needed to accomplish these goals is empirical data, public input, and engagement with experts from all relevant fields—and, more specifically, multiple experts from each field given that they have diverse opinions and experiences related to IGG that no one person from their field can fairly represent. Our study demonstrates the feasibility and value of systematically engaging with a large and heterogeneous group of such experts to deliberate on a policy agenda for IGG.

## Supporting information

S1 Survey Questionnaire in round 2 survey. (PDF)

S1 Table IGG practices rejected as top priority in round 2. ^a^ Short labels were not provided in the round 2 survey. See Table 2 for each practice’s full descriptive statement.
(PDF)
