# Supplementary material for: Investigative genetic genealogy practices warranting policy attention: Results of a modified policy Delphi
Source: PLoS Genet. 2025 Jan 16;21(1):e1011520. doi: 10.1371/journal.pgen.1011520 (PMC11737847; doi:10.1371/journal.pgen.1011520)
Supplement: S1 Survey — (PDF) [file pgen.1011520.s001.pdf]

S1 Survey

Thank you for participating in this Delphi study on investigative genetic genealogy (IGG).

In this second Delphi round, we are asking you to respond to and prioritize practices related to IGG that were identified as problematic to some participants during the first Delphi round, which was the April virtual meeting.

Your participation in this survey is voluntary and you may end the survey at any time. Your refusal to participate will involve no penalty or loss of benefits to which you might otherwise be entitled. Your responses will be kept confidential. No individually identifiable information will be reported unless you ask for something to be attributed specifically to you. There are no reasonably foreseeable benefits to you of participating in the survey, although we hope the results will be broadly useful in identifying problematic practices related to IGG and generating potential policy options to address them.

The survey will take approximately 45 minutes to complete. You do not need to complete it in one sitting. You can stop and return to it whenever it is convenient for you. Your responses will be saved as you progress. Please feel free to take your time.

Finally, we strongly recommend that you complete this survey on a laptop, desktop, or tablet rather than a mobile phone. Due to the formatting of the survey, it may be challenging to select answers on a mobile phone.

If you are willing to participate, please click the forward arrow below. Proceeding with the survey will constitute your consent to participate.

We are interested in your perspectives on practices related to IGG that warrant policy attention.

In this survey, we define **"practices"** broadly to include current activities and conditions as well as possible future activities and conditions.

We define **"practices that warrant policy attention"** as practices that should be prioritized for policy intervention because they affect, or could affect, individuals or society in unacceptable ways.

We define **"policy"** broadly as any kind of law, regulation, rule, contractual term, standard, or guideline developed by a governmental, institutional, commercial, or professional entity that is intended to influence behavior. Policies can influence behavior by requiring, prohibiting, encouraging, or discouraging specific conduct.

In later Delphi rounds, we will ask for your opinions on specific policy options. In this Delphi round, we want you to focus on identifying practices that should be prioritized for policy attention.

As you may recall from the first Delphi round, we organized practices related to IGG according to four domains:

1. Case eligibility and selection
2. Consent and notification
3. Data management, privacy, and security
4. Governance and accountability

In this survey, we will introduce each domain with a background statement and summary. We will then ask you to consider specific practices related to the domain. For each practice, we will ask you to categorize the practice according to whether you believe it warrants **high, medium, low, or no priority** for policy attention. Please select high, medium, or low priority if you view the practice as problematic and in need of policy attention. Select no priority if you view the current practice as acceptable and not in need of policy attention.

You will be allowed to respond that you are unable to assign a priority category to a particular practice.

However, we ask that you select this option sparingly and only when you either have no knowledge of the practice or it is inaccurate or so confusing as presented that you cannot make sense of it.

At the end of the survey, we will ask you to select up to three practices that you believe warrant the highest policy attention. Then, we will ask whether you oppose or favor certain practices.

Let's start with an example.

Let's pretend that four individuals named Zach, Miranda, Kit, and Sasha are on the Delphi panel with you and are taking this survey.

After reading the background statement and summary for the first domain, which is case eligibility and selection, they come to the following practice:

Law enforcement can generally use IGG in investigations of active cases and does not need to wait until all other techniques have been attempted and failed and cases have gone cold.

How do you prioritize this practice for policy attention?

- (●) High priority
- ( ) Medium priority
- ( ) Low priority
- ( ) No priority
- ( ) Unable to prioritize (please explain)

Zach's selection is shown above. Zach believes that IGG should be reserved for cold cases only and feels strongly that policies are needed to change this practice. He categorizes it as "**high priority**" for policy attention.

Miranda also believes that IGG should be reserved for cold cases. Although Miranda believes it might be a good idea for policies to discourage use of IGG in active cases, she does not feel strongly about this. She categorizes the practice as "**low priority**" for policy attention.

Kit agrees that IGG should be allowed in active cases. However, Kit believes it is important for policies to clarify that investigators may use IGG in active cases. They categorize the practice as "**high priority**" for policy attention.

Sasha agrees that IGG should be allowed in active cases but does not think any policies related to the practice are needed or might be a good idea at this time. She categorizes the practice as "**no priority**" for policy attention.

Now it’s your turn. Let’s begin with the first domain. Here is the background statement and summary. As a reminder, these background statements are largely based on your discussions during the virtual meeting in April.

Domain 1: Case eligibility and selection

IGG is an investigative technique that can be used to help identify criminal perpetrators and human remains using their DNA. IGG is effective and efficient, but it requires time and money. Because law enforcement has limited resources, not every case can be investigated using IGG. The anticipated costs and likelihood of success of using IGG are specific to each case under investigation.

Some laws, policies, and database rules restrict the use of IGG to certain kinds of cases. For example, the policies of FamilyTreeDNA and GEDmatch, which are two major databases that allow IGG, restrict its use to investigations of certain violent crimes and human remains. However, they identify different eligible crimes. For example, FamilyTreeDNA identifies eligible crimes to include sexual assault, whereas GEDmatch includes aggravated rape.

Another issue is that states have adopted different definitions for eligible crimes. For example, each state defines sexual assault to include a different set of unwanted sexual activities. Some question whether certain non-violent crimes, such as burglary of unoccupied homes, should be eligible for IGG or, more generally, whether there should be an opportunity to recognize case eligibility exceptions. To illustrate, the crime of stalking is not eligible for IGG, but an exception to this rule might be justified when the crime continues for many years and includes specific and credible threats of harm to victims and their family.

Both databases allow IGG to help identify abandoned, deceased newborns (“Baby Doe” cases), although some believe that IGG should not be used to investigate these cases because they might result in unjustified or disproportionate prosecution of women who might have been vulnerable at the time of pregnancy and abandonment. At the same time, IGG might be underutilized in cases involving wrongly accused persons and victims from marginalized communities, including victims of color and victims engaged in high-risk activities, such as sex workers. Additionally, although it is technically possible to use IGG at any point in an investigation, and in some cases is being used alongside other techniques, some believe that it should only be used as a last resort after all other techniques have been attempted but failed and the case has gone cold.

Finally, the identity of the person under investigation typically is confirmed by directly comparing STR profiles from the unknown sample DNA with DNA taken directly from the individual. When the unknown sample DNA cannot support a direct comparison of STR profiles, however, it is unclear if or how IGG should proceed.

We will now present 10 practices related to **case eligibility and selection**. We will ask you to categorize each according to whether, in your opinion, it warrants high, medium, low, or no priority for policy attention.

As a reminder, we define “practices” broadly to include current and future activities and conditions. In some cases, a practice might describe a state of affairs.

Each database has identified a different set of IGG-eligible crimes.

How do you prioritize this practice for policy attention?

- ○ High priority
- ○ Medium priority
- ○ Low priority
- ○ No priority
- ○ Unable to prioritize (please explain): [free text]

States have adopted different definitions for IGG-eligible crimes.

How do you prioritize this practice for policy attention?

- ○ High priority
- ○ Medium priority
- ○ Low priority
- ○ No priority
- ○ Unable to prioritize (please explain): [free text]

Database rules and other policies currently restrict IGG to violent crimes and do not permit its use for non-violent crimes.

How do you prioritize this practice for policy attention?

- ○ High priority
- ○ Medium priority
- ○ Low priority
- ○ No priority
- ○ Unable to prioritize (please explain): [free text]

Database rules and other policies do not currently allow any exceptions to case eligibility criteria.

How do you prioritize this practice for policy attention?

- ○ High priority
- ○ Medium priority
- ○ Low priority
- ○ No priority
- ○ Unable to prioritize (please explain): [free text]

IGG is used by defense and post-conviction attorneys for the purpose of exonerating persons wrongly accused of or convicted for crimes less frequently than by law enforcement and prosecutors.

How do you prioritize this practice for policy attention?

- ○ High priority
- ○ Medium priority
- ○ Low priority
- ○ No priority
- ○ Unable to prioritize (please explain): [free text]

IGG is used to investigate cases involving victims from marginalized communities less frequently than those involving other victims, especially victims of European descent.

How do you prioritize this practice for policy attention?

- ○ High priority
- ○ Medium priority
- ○ Low priority
- ○ No priority
- ○ Unable to prioritize (please explain): [free text]

Use of IGG to identify Baby Does has led to prosecution of mothers that might be unjustified or disproportionate.

How do you prioritize this practice for policy attention?

- ○ High priority
- ○ Medium priority
- ○ Low priority
- ○ No priority
- ○ Unable to prioritize (please explain): [free text]

Law enforcement can generally use IGG in investigations of active cases and does not need to wait until all other techniques have been attempted and failed and cases have gone cold.

How do you prioritize this practice for policy attention?

- ○ High priority
- ○ Medium priority
- ○ Low priority
- ○ No priority
- ○ Unable to prioritize (please explain): [free text]

Law enforcement might not sufficiently or consistently take into account the costs of IGG, relative to the likelihood of its success, when deciding whether to use IGG in specific cases.

How do you prioritize this practice for policy attention?

- ○ High priority
- ○ Medium priority
- ○ Low priority
- ○ No priority
- ○ Unable to prioritize (please explain): [free text]

It is possible to conduct IGG even when a direct comparison of STR profiles to confirm identity is not possible.

How do you prioritize this practice for policy attention?

- ○ High priority
- ○ Medium priority
- ○ Low priority
- ○ No priority
- ○ Unable to prioritize (please explain): [free text]

Is there anything you would like to share about **case eligibility and selection** for IGG? [free text]

Domain 2: Consent and notification

GEDmatch and FamilyTreeDNA have adopted different default approaches to obtaining consent for IGG from database participants. Their approaches have changed over time, but currently, one database opts all U.S. participants in to IGG, and the other database opts all participants out, although both databases allow participants to change this setting. Some question whether a default opt-in consent approach is sufficient to promote participant autonomy. Moreover, the consent forms have been criticized as long and complex, causing some to doubt whether the consent that is provided is truly informed. Regardless of their default approach, both databases obtain broad consent to all instances of IGG rather than specific consent to each instance of IGG.

Although it appears that law enforcement typically respects the consent selections of database participants when they conduct IGG, they are not always legally required to do so. Some object to law enforcement participation in databases against terms of service on grounds that it undermines individual privacy interests and might weaken public support; others are not concerned about the practice given its similarity to other legal, investigative techniques.

Currently, neither database notifies database participants when they have been identified as a genetic relative of an unknown DNA sample uploaded by law enforcement. Some believe this information should be disclosed, or at least offered, while others view such disclosure as unnecessary or intrusive.

Importantly, the databases restrict their consent practices to database participants. They do not attempt to obtain consent from, nor do they require participants to obtain consent from, genetic relatives who are not database participants but are identified from the family tree built during IGG. If such a practice were to be implemented, it is unclear which relatives should be asked for consent, or how.

Finally, consent is relevant to reference testing, which involves law enforcement collecting and testing DNA

from individuals on the family tree who are not database participants to help them narrow their investigation. Typically, law enforcement obtains reference DNA from a reference tester with their knowledge and consent. However, some policies permit law enforcement to surreptitiously collect and test reference DNA if asking the reference tester for consent would compromise the investigation.

We will now present 8 practices related to **consent and notification**. As with the first domain, we will ask you to categorize each practice according to whether, in your opinion, it warrants high, medium, low, or no priority for policy attention.

Databases have adopted different default consent approaches to IGG: one opts U.S. database participants in to IGG and the other opts all participants out.

How do you prioritize this practice for policy attention?

- ○ High priority
- ○ Medium priority
- ○ Low priority
- ○ No priority
- ○ Unable to prioritize (please explain): [free text]

Databases do not obtain specific consent to each instance of IGG, but rather they obtain broad consent to IGG in all cases.

How do you prioritize this practice for policy attention?

- ○ High priority
- ○ Medium priority
- ○ Low priority
- ○ No priority
- ○ Unable to prioritize (please explain): [free text]

Database consent forms can be long and complex, which might interfere with database participants' comprehension.

How do you prioritize this practice for policy attention?

- ○ High priority
- ○ Medium priority
- ○ Low priority
- ○ No priority
- ○ Unable to prioritize (please explain): [free text]

Databases can change their policies and terms of service at any time.

How do you prioritize this practice for policy attention?

- ○ High priority
- ○ Medium priority
- ○ Low priority
- ○ No priority
- ○ Unable to prioritize (please explain): [free text]

Law enforcement is generally allowed to participate in databases against terms of service and to disregard the consent selections of database participants when they conduct IGG.

How do you prioritize this practice for policy attention?

- ○ High priority
- ○ Medium priority
- ○ Low priority
- ○ No priority
- ○ Unable to prioritize (please explain): [free text]

Databases currently do not notify, or offer to notify, database participants when they are identified as a genetic relative of the unknown DNA sample.

How do you prioritize this practice for policy attention?

- ○ High priority
- ○ Medium priority
- ○ Low priority
- ○ No priority
- ○ Unable to prioritize (please explain): [free text]

Databases do not currently obtain consent to IGG, or require participants to obtain consent to IGG, from genetic relatives of database participants who are not themselves database participants.

How do you prioritize this practice for policy attention?

- ○ High priority
- ○ Medium priority
- ○ Low priority
- ○ No priority
- ○ Unable to prioritize (please explain): [free text]

Law enforcement is generally allowed to collect reference samples surreptitiously and without consent from reference testers.

How do you prioritize this practice for policy attention?

- ○ High priority
- ○ Medium priority
- ○ Low priority
- ○ No priority
- ○ Unable to prioritize (please explain): [free text]

Is there anything you would like to share about **consent and notification** for IGG? [free text]

Domain 3: Data management, privacy, and security

IGG involves the management and analysis of information, including genetic information, that some view as highly sensitive. In particular, during IGG, a SNP profile is developed from the unknown DNA sample and uploaded to one or more genetic genealogy databases. Unlike STR profiles that are uploaded to law enforcement databases, SNP profiles can reveal a person's nonvisible traits and their current and future risk of medical conditions. To minimize intrusions on individual privacy, some laws and policies forbid law enforcement from accessing and using this information in investigations, but these restrictions are not universal. Further, some are concerned that the security practices and policies of databases do not sufficiently protect customer information, including SNP profiles, from inadvertent disclosure or unauthorized access, citing recent hacking incidents.

More generally, it is unclear how law enforcement, independent genetic genealogists, and private laboratories should record, store, and transfer various kinds of information generated from IGG. In addition, there is uncertainty regarding when, exactly, SNP profiles should be removed from genetic genealogy databases during the course of an investigation. There is also some debate regarding whether and when DNA samples collected during IGG, and genetic information and family trees generated during IGG, should be destroyed. Law enforcement is obligated to preserve certain evidence, but information generated during IGG typically is not viewed by law enforcement as evidence. Moreover, preservation of information about non-suspect genetic relatives could undermine their privacy.

Finally, some are concerned that law enforcement might use DNA samples involved in IGG for other purposes, such as to populate an unregulated law enforcement database, or attempt to conduct IGG in medical or research databases. Separately, some are concerned that laboratories might reuse these samples and related data for commercial or research purposes.

We will now present 7 practices related to **data management, privacy, and security**. Please categorize each according to whether you think it warrants high, medium, low, or no priority for policy attention.

Practices for recording, storing, and transferring DNA samples and related data collected and generated during IGG are inconsistent and might not be sufficiently protective of individual privacy.

How do you prioritize this practice for policy attention?

- ○ High priority
- ○ Medium priority
- ○ Low priority
- ○ No priority
- ○ Unable to prioritize (please explain): [free text]

Practices for preserving information developed during IGG are inconsistent and might undermine individual privacy.

How do you prioritize this practice for policy attention?

- ○ High priority
- ○ Medium priority
- ○ Low priority
- ○ No priority
- ○ Unable to prioritize (please explain): [free text]

Genetic genealogy databases might not be sufficiently secure to prevent unauthorized access to or disclosure of information in the database.

How do you prioritize this practice for policy attention?

- ○ High priority
- ○ Medium priority
- ○ Low priority
- ○ No priority
- ○ Unable to prioritize (please explain): [free text]

Law enforcement might use medical information from SNP profiles developed during IGG in their investigations.

How do you prioritize this practice for policy attention?

- ○ High priority
- ○ Medium priority
- ○ Low priority
- ○ No priority
- ○ Unable to prioritize (please explain): [free text]

Law enforcement might conduct IGG in medical and research databases.

How do you prioritize this practice for policy attention?

- ○ High priority
- ○ Medium priority
- ○ Low priority
- ○ No priority
- ○ Unable to prioritize (please explain): [free text]

Law enforcement might use data from DNA samples collected during IGG to populate unregulated law enforcement databases.

How do you prioritize this practice for policy attention?

- ○ High priority
- ○ Medium priority
- ○ Low priority
- ○ No priority
- ○ Unable to prioritize (please explain): [free text]

Laboratories that develop SNP profiles for IGG might reuse DNA samples and related data for other purposes.

How do you prioritize this practice for policy attention?

- ○ High priority
- ○ Medium priority
- ○ Low priority
- ○ No priority
- ○ Unable to prioritize (please explain): [free text]

Is there anything you would like to share about **data management, privacy, and security**? [free text]

You are almost done with the survey! This is the last domain to consider.

Domain 4: Governance and accountability

IGG is currently governed by a patchwork of federal guidance, state laws, database policies, professional guidelines, and best practices. Although patchwork governance promotes tailoring solutions to different problems, settings, and locations, different mechanisms might be inconsistent with one another, which can create confusion for those seeking to comply with them. Additionally, compliance with some governance mechanisms is advisory and therefore not required, and those that are mandatory might not include sufficient penalties to ensure compliance. More generally, there are few remedies available to those who are harmed by IGG, such as genetic relatives of the person whose identity is under investigation. Such harms might result from disclosure of their personal information that is collected during IGG. To minimize harms and promote accountability, it is possible to require judicial oversight of IGG, although this could add burden on investigators without sufficient offsetting benefits.

While some prefer comprehensive laws for IGG, others do not believe this is necessary at this time. This second group might include those who believe expert groups of IGG practitioners, end users, and facilitators should take a leadership role in governance and develop guidelines and best practices for practitioners. This second group also might include those who endorse relatively narrow laws that target some, but not all, IGG actors and activities. Genetic genealogists and private laboratories have been identified as two actors involved in IGG whose activities are currently underregulated. In particular, some believe that regulation of these actors is necessary to address concerns about the quality of their work specific to IGG.

Finally, public information about uses and outcomes of IGG is currently limited. Some believe that this

information can promote accountability, while others question its value and note technical difficulties with compliance.

We will now present 8 practices related to **governance and accountability**. Please categorize each according to whether you think it warrants high, medium, low, or no priority for policy attention.

IGG currently is governed by a patchwork of laws, guidelines, policies, and best practices, rather than a standardized governance framework.

How do you prioritize this practice for policy attention?

- ○ High priority
- ○ Medium priority
- ○ Low priority
- ○ No priority
- ○ Unable to prioritize (please explain): [free text]

In some jurisdictions, IGG is governed largely by guidelines, best practices, and other self-regulatory mechanisms developed for IGG by expert groups.

How do you prioritize this practice for policy attention?

- ○ High priority
- ○ Medium priority
- ○ Low priority
- ○ No priority
- ○ Unable to prioritize (please explain): [free text]

Judicial oversight of IGG is not required in most jurisdictions.

How do you prioritize this practice for policy attention?

- ○ High priority
- ○ Medium priority
- ○ Low priority
- ○ No priority
- ○ Unable to prioritize (please explain): [free text]

Some governance mechanisms might not include sufficient penalties for noncompliance.

How do you prioritize this practice for policy attention?

- ○ High priority
- ○ Medium priority
- ○ Low priority
- ○ No priority
- ○ Unable to prioritize (please explain): [free text]

There are limited remedies available to those who are harmed by IGG.

How do you prioritize this practice for policy attention?

- ○ High priority
- ○ Medium priority
- ○ Low priority
- ○ No priority
- ○ Unable to prioritize (please explain): [free text]

Genetic genealogists are generally not subject to governance mechanisms that promote quality, such as licensing requirements.

How do you prioritize this practice for policy attention?

- ○ High priority
- ○ Medium priority
- ○ Low priority
- ○ No priority
- ○ Unable to prioritize (please explain): [free text]

Private laboratories are subject to few governance mechanisms that promote SNP profile quality, such as requirements regarding use of validated techniques.

How do you prioritize this practice for policy attention?

- ○ High priority
- ○ Medium priority
- ○ Low priority
- ○ No priority
- ○ Unable to prioritize (please explain) [free text]

Information about IGG uses and outcomes is generally not collected and shared with the public.

How do you prioritize this practice for policy attention?

- ○ High priority
- ○ Medium priority
- ○ Low priority
- ○ No priority
- ○ Unable to prioritize (please explain): [free text]

Is there anything you would like to share about **governance and accountability** for IGG? [optional free-text response]

Only two sets of questions left in the survey.

Highest priority

Now, we will show you all of the practices, across all of the domains, that you categorized as "**high priority**" for policy attention.

Please check the box for up to three practices that you believe should receive the highest priority.

[only practices selected earlier as "high priority" are shown]

In this last section, we will ask if you oppose or favor certain practices.

What is your view of this practice?

	Strongly oppose	Somewhat oppose	Neutral	Somewhat favor	Strongly favor
Use of IGG to investigate non-violent crimes	○	○	○	○	○
Use of IGG to investigate active cases before all other techniques have been attempted and failed and the cases have gone cold	○	○	○	○	○
Use of IGG to identify Baby Does	○	○	○	○	○
Use of IGG when an STR profile cannot be developed from the unknown DNA sample and so cannot be used to confirm identity	○	○	○	○	○
Default consent approach to IGG that opts all participants in to IGG	○	○	○	○	○
Default approach of obtaining consent to all eligible uses of IGG rather than consent to each specific use	○	○	○	○	○

What is your view of this practice?

	Strongly oppose	Somewhat oppose	Neutral	Somewhat favor	Strongly favor
Law enforcement participation in databases against terms of service	○	○	○	○	○
Notification to database participants when they are identified as a genetic relative of an unknown DNA sample during IGG	○	○	○	○	○
Surreptitious collection/testing of DNA from suspects to confirm their identity	○	○	○	○	○
Surreptitious collection/testing of DNA from genetic relatives who are not suspects to help focus an investigation	○	○	○	○	○
Laboratory reuse of DNA samples collected and tested during IGG for research purposes	○	○	○	○	○
Use of IGG only with specific permission from a court and subject to its oversight	○	○	○	○	○

What is your view of this practice?

	Strongly oppose	Somewhat oppose	Neutral	Somewhat favor	Strongly favor
Mandatory licensure of genetic genealogists who conduct/facilitate IGG	○	○	○	○	○
Regulation of IGG via federal law	○	○	○	○	○
Regulation of IGG via individual state laws	○	○	○	○	○
Regulation of IGG via best practices and guidelines developed by IGG practitioners/stakeholders	○	○	○	○	○

Is there anything you would like to share about the practices you most **strongly oppose**, most **strongly favor**, and/or you believe warrant the **highest priority** for policy attention? [free text]]
