# Supplementary material for: Investigative genetic genealogy practices warranting policy attention: Results of a modified policy Delphi
Source: PLoS Genet. 2025 Jan 16;21(1):e1011520. doi: 10.1371/journal.pgen.1011520 (PMC11737847; doi:10.1371/journal.pgen.1011520)
Supplement: S1 Table — a Short labels were not provided in the round 2 survey. See Table 2 for each practice’s full descriptive statement. (PDF) [file pgen.1011520.s002.pdf]

S1 Table. IGG practices rejected as top priority in round 2

Practice, short label[a]	Criteria used for prioritization				Other counts (not used for prioritization)			
	General priority score	Avg priority score	Highest priority count	High priority count	Medium priority count	Low priority count	No priority count	Unable to prioritize count
Limited oversight of genetic genealogists	91	2.9	4	14	6	6	5	0
Law enforcement use of medical data	86	2.9	4	10	9	8	3	1
Insufficient penalties for non-compliance	97	3.1	3	14	8	8	1	0
Surreptitious collection of reference samples	83	3.0	3	11	9	4	4	3
Database policy changes	80	2.7	3	10	5	10	5	1
Security vulnerabilities of databases	102	3.3	2	15	11	4	1	0
Different database consent approaches	86	3.0	2	12	7	7	3	2
Self-regulation of IGG	97	3.1	2	11	15	3	2	0
IGG restriction to violent crimes	74	2.5	2	7	7	9	7	1
IGG use in active investigations	79	2.7	1	10	5	10	4	2
Different database eligibility criteria	78	2.8	1	9	6	11	2	3
No judicial oversight of IGG	76	2.7	1	8	8	8	4	3
No exceptions to eligibility criteria	88	2.8	1	7	14	8	2	0
Complex consent forms	71	2.5	1	4	11	9	4	3
No consent from genetic relatives not participating in databases	52	1.9	1	3	5	6	13	4
Inconsistent/underuse of cost-benefit analysis	69	2.3	1	2	8	17	3	1
Inconsistent/inadequate data preservation	104	3.5	0	16	12	2	0	1
IGG underuse for victims from marginalized communities	86	3.2	0	12	10	3	2	4
Limited remedies for persons harmed by IGG	79	2.9	0	10	5	12	0	4
Limited transparency about IGG uses/outcomes	81	2.8	0	8	8	12	1	2
Different state crime definitions	78	2.8	0	8	10	6	4	3
IGG use in Baby Doe cases	74	2.6	0	8	7	8	5	3
No specific consent	65	2.2	0	5	6	8	11	1
No notification to database participants	61	2.1	0	4	4	12	9	2

[a] Short labels were not provided in the round 2 survey. See Table 2 for each practice's full descriptive statement.
